# Evolution shapes the responsiveness of the D-box enhancer element to light and reactive oxygen species in vertebrates

**DOI:** 10.1038/s41598-018-31570-8

**Published:** 2018-09-04

**Authors:** Cristina Pagano, Rima Siauciunaite, Maria L. Idda, Gennaro Ruggiero, Rosa M. Ceinos, Martina Pagano, Elena Frigato, Cristiano Bertolucci, Nicholas S. Foulkes, Daniela Vallone

**Affiliations:** 1 Institute of Toxicology and Genetics, Karlsruhe Institute of Technology, Eggenstein-Leopoldshafen, Germany; 2 Department of Life Sciences and Biotechnology, University of Ferrara, Ferrara, Italy; 3 Present Address: CNR, ISASI “E. Caianiello” Pozzuoli, Naples, Italy; 4 Present Address: Laboratory of Genetics and Genomics, National Institute on Aging Intramural Research Program, National Institutes of Health, Baltimore, Maryland USA; 5 Present Address: Facultade de Bioloxía, Universidade de Vigo, Vigo, Spain; 6 Present Address: Department of Biochemistry, Biophysics and General Pathology, University of Campania “Luigi Vanvitelli” Naples, Naples, Italy

## Abstract

The circadian clock is a highly conserved cell-autonomous mechanism that directs daily rhythms in most aspects of biology. Daily entrainment by environmental signals, notably light, is essential for its function. However, our understanding of the mechanisms and the evolution of photic entrainment remains incomplete. Fish represent attractive models for exploring how light regulates the circadian clock due to the direct light sensitivity of their peripheral clocks. Central to this property is the light induced expression of clock genes that is mediated by D-box enhancer elements. Here, using zebrafish cells, we reveal that the light responsive D-box enhancer serves as a nuclear target for reactive oxygen species (ROS). We demonstrate that exposure to short wavelengths of visible light triggers increases in ROS levels via NADPH oxidase activity. Elevated ROS activates the JNK and p38 MAP kinases and in turn, induces clock gene expression via the D-box. In blind cavefish and mammals, where peripheral clocks are no longer entrained by direct illumination, ROS levels are still increased upon light exposure. However, in these species ROS no longer induces D-box driven clock gene transcription. Thus, during evolution, alterations in ROS-responsive signal transduction pathways underlie fundamental changes in peripheral clock photoentrainment.

## Introduction

The circadian clock is a highly conserved biological timing mechanism shared by most organisms from cyanobacteria to humans. It has evolved to anticipate the regular environmental changes associated with the day-night cycle and thereby coordinates physiological and behavioral adaptations required for survival^1,2^. At its simplest level, the circadian clock can be considered to be composed of a pacemaker that generates rhythmicity, an input pathway that resets the clock on a daily basis in response to environmental signals (zeitgebers) that are indicative of the time of day and, finally, an output pathway through which the circadian clock conveys timing information to regulate physiology and behavior^3^.

At the anatomical level, the vertebrate circadian clock consists of central pacemakers (e.g. the suprachiasmatic nucleus (SCN) in the hypothalamus) and of multiple independent peripheral clocks distributed in most tissues, organs and cells. Central pacemakers coordinate peripheral clocks via a complex combination of systemic signals^4–6^. Light input to the clock in mammals occurs exclusively through the retina, via a subset of intrinsically photosensitive retinal ganglion cells (ipRGCs) which express the non-visual photoreceptor, melanopsin^7–9^. Signals from these cells are conveyed indirectly to the entire circadian timing system, via the retinohypothalamic tract and the SCN^3,10^. However, in certain non-mammalian vertebrates, notably fish, direct exposure of tissues and cells to light leads to entrainment of the local peripheral clocks^11^.

At the molecular level, the circadian clock consists of transcription–translation autoregulatory feedback loops^12^. In vertebrates, the positive elements of these regulatory circuits are the BMAL and CLOCK basic helix–loop–helix (bHLH), Per-Arnt-Single minded (PAS) transcription factors. These proteins bind as heterodimeric complexes to canonical E-box enhancer elements (5′-CACGTG-3′) present in the promoter regions of the negative elements of the circuit (the period Per, and cryptochrome Cry, families) or in clock controlled genes^13,14^. Following transcriptional activation of the *per* and *cry* genes and their translation, PER and CRY heterodimerize, translocate from the cytoplasm to the nucleus and then inhibit their own transcription by interacting with and inhibiting transcriptional activation by CLOCK and BMAL^15^. Additional feedback loops serve to stabilize this core loop which completes one cycle in *circa* 24 hours^16^. In the majority of organisms, light represents the most potent zeitgeber and specialized mechanisms have evolved for the detection of daily changes in its intensity as well as spectrum^17,18^. In the case of vertebrates, considerable attention has been placed on the function of the circadian photoreceptor, melanopsin and in particular, the membrane-associated signalling events that underlie its function^8^. However, a more general understanding of how light-triggered signal transduction pathways impact upon gene expression and in particular how these pathways have been shaped over the course of vertebrate evolution remains very much incomplete.

Close links exist between the circadian clock and oxidative stress. It has been speculated that during the origin of life on earth, one of the first driving forces for the evolution of the circadian clock was the great oxidation event that occurred following the development of photosynthetic bacteria and the photo-dissociation of water^19^. The evolution of an internal 24 hours timing mechanism enabled the anticipation of a day night cycle in oxidative stress and thereby permitted a temporally coordinated homeostatic response. In addition, redox state has been shown to serve as a signal for entraining the circadian clock in a range of model organisms^20,21^. This regulation has been predicted to serve as a bridge between metabolism and the circadian timing system, thereby enabling the clock to respond to changes in metabolic activity^22^. However, excess oxidative stress can also result in the damage of nucleic acids, proteins and lipids, and has been implicated in various pathologies^23^. Therefore, many questions remain concerning how elevated ROS levels are interpreted intracellularly as a clock regulating signal rather than a stressor.

The zebrafish, *Danio rerio*, has become a powerful model for exploring how various environmental factors impact upon the circadian clock. Zebrafish possess directly light entrainable peripheral circadian clocks^11,24,25^. Direct illumination of zebrafish tissues or even cell lines results in the activation of a subset of clock genes that, in turn, leads to local circadian clock entrainment. Previously, we have demonstrated that the D-box enhancer serves as the primary element driving light-dependent clock gene transcription^26,27^. In addition, in a comparative study using the zebrafish and a blind cavefish species (*Phreatichthys andruzzii)*, where light entrainment of the clock has been lost during evolution, we have demonstrated that at least two non-visual opsins (TMT-opsin and Melanopsin (Opn4m2)), play a role in the light sensing mechanism of fish peripheral clocks^28^. However, our understanding of the precise mechanisms underlying the photic regulation of these peripheral clocks remains incomplete. Indeed, functional genomic analysis in zebrafish has identified more than 40 opsins of which 32 are non-visual opsins expressed in peripheral organs^17,29^. Furthermore, other non-opsin based photoreceptor systems, have also been implicated in peripheral photoreception in zebrafish, including flavin-containing oxidases which generate ROS species upon exposure to light. In particular, a study of light-regulated peripheral clock entrainment in the zebrafish embryonic cell line Z3, revealed that light-driven increases in intracellular ROS levels activate clock gene expression^30^. While ERK/MAP kinase signalling was implicated as an essential positive element in the context of light and ROS-dependent clock gene expression^30,31^, the enzymatic function of the antioxidant enzyme, Catalase, was shown to serve as a negative regulator^30^. However, many questions remain concerning which class of flavin-containing oxidases is able to transduce light signals into the elevation of ROS levels, as well as precisely which signalling pathways and promoter elements mediate ROS-driven clock gene expression.

A major step during vertebrate evolution has been the transition from directly light regulated peripheral clocks in groups such as fish, to the centralized, retina-based photoreception system observed in modern mammals^3^. These major differences in the circadian timing system predict that alterations in the regulatory networks of peripheral clock input pathways must have occurred over the course of vertebrate evolution. Whether these events have occurred at the level of photoreceptors, signal transduction pathways or transcriptional regulatory mechanisms remains poorly understood.

Here, we demonstrate that in zebrafish cells, the accumulation of ROS species triggered by blue light is a prerequisite step for light-regulated D-box-driven gene expression. This ROS production, driven by NOX-NADPH oxidase proteins, is associated with the rapid, and transient induction of the JNK and p38 stress-activated MAP kinase pathways. In a comparative study, we explored the fate of key steps of this signalling pathway in species which have lost directly light entrainable peripheral clocks during evolution. In both the blind cavefish *P. andruzzii* and mammalian cells, similar to the situation in zebrafish, blue light triggers an increase in cellular ROS levels as well as activation of the MAP kinase pathways. However, subsequently these events do not result in activation of D-box enhancer mediated clock gene transcription. This reveals that evolution of the photoentrainment pathway in vertebrate peripheral tissues has acted at multiple levels, involving not only changes in photoreceptor function, but also affecting downstream signalling elements.

## Results

### Entrainment of the clock and clock gene activation by H_2_O_2_

**Figure 1:**
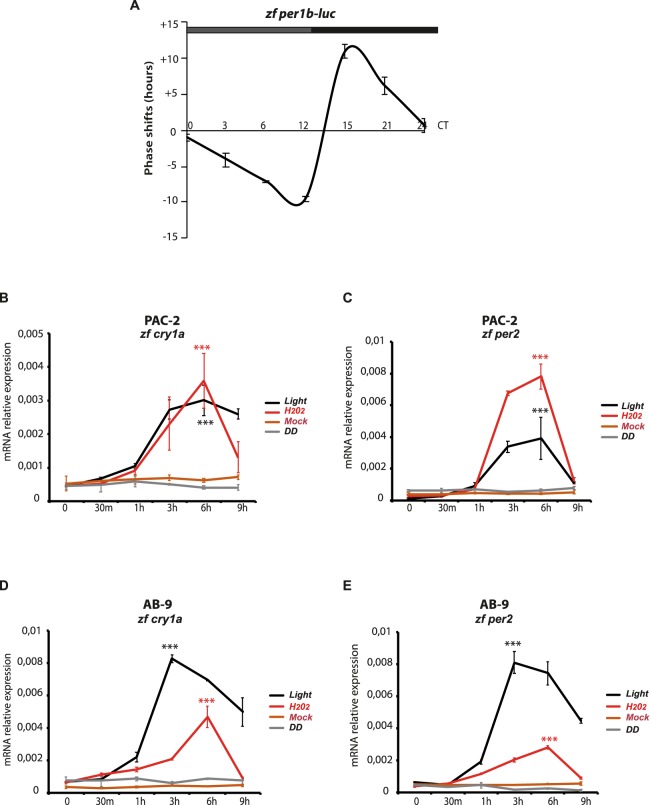
H_2_O_2_ entrains the zebrafish cell circadian clock. (**A**) Phase response curve (PRC) analysis of the effects of H_2_O_2_ treatment delivered at different circadian time points (CT) on rhythmic *zf per1b-Luc* expression in PAC-2 cells. Means of phase shifts ± SD (n = 4–8) are plotted on the y*-*axis (negative and positive values correspond to phase delays and advances, respectively). Circadian time is plotted on the x-axis. See also Fig. S1 for original bioluminescence rhythms. (**B**–**E**) qRT-PCR analysis of *zfcry1a* and *zfper2* expression in PAC-2 (**B**,**C**) and AB-9 (**D**,**E**) cells. Samples were taken at different time points after addition of H_2_O_2_ (red traces) or exposure to light (black traces). Grey and brown traces indicated control samples maintained in constant darkness (DD) or mock treated (L15 medium), respectively. Mean mRNA relative expression (n = 3) ± SD is plotted on the y-axes, whereas time is plotted on the x-axes. Levels of significance between peak points of expression and time 0 are calculated by t-test and are indicated (***p < 0.001, **p < 0.01, *p < 0.05).

A previous report has linked light induced ROS levels with the activation of clock gene expression in the zebrafish Z3 cell line^30^. In order to explore in more detail, the links between ROS and the core clock machinery, we first tested whether ROS induction resets the phase of a previously light cycle-entrained circadian clock in an independent zebrafish embryo-derived cell line, PAC-2. We chose to monitor the effect of H_2_O_2_ treatment on our bioluminescent clock reporter PAC-2 cell line where a luciferase reporter gene is stably expressed under the transcriptional control of the *zfper1b* promoter^25^. The *per1b-luc* expressing cells were synchronized by exposure to light-dark cycles (LD, 12/12 hr) and then transferred to constant darkness (DD) where the bioluminescence rhythms persist for several cycles under free-running conditions. On the first day of this free running period, 300 µM H_2_O_2_ was added to different groups of cells, each group at different circadian times (CT, where CT 0 and CT 12 are defined as the times when the light would normally be turned on and off, respectively). The bioluminescence rhythm of each group was monitored and compared with that of an untreated control cell group in order to plot a Phase Responsive Curve (PRC) (Figs 1A and S1). Consistent with H_2_O_2_ serving as a signal for entraining the circadian clock, H_2_O_2_ was able to adjust the phase of the bioluminescence rhythm as a function of the time of its addition. H_2_O_2_ treatment during the subjective day resulted in a phase delay in the *zf per1b-luc* expression rhythm, while treatment during the subjective night lead to a phase advance. Instead, no significant phase shift was observed upon H_2_O_2_ treatment at CT 0 and CT 24. This result closely resembles the entraining effects of light previously documented by our group for the PAC-2 cell line^25^, where maximum phase shifts were observed for light pulses delivered at the light-dark transition.

Many previous studies have implicated the acute induction of *zfcry1a* and *zfper2* as a key step in the entrainment of the circadian clock mechanism by light^32,33^. Using qRT- PCR analysis in PAC-2 cells we investigated whether these light inducible clock genes were also induced upon H_2_O_2_ treatment. Cells were maintained in constant darkness for at least three days and then acutely treated with 300 µM H_2_O_2_ or with L15 medium (mock). RNA samples were then harvested at different time points during a 9 hours period. As a positive and negative control for activation of the expression for both genes, a set of samples exposed acutely to white light or maintained in DD, were also harvested simultaneously (Fig. 1B,C). Consistent with previous reports^30^, the expression of *zfcry1a* and *zfper2* was increased by H_2_O_2_ treatment (red traces) during the first 6 hours followed by a rapid decrease with kinetics similar to those observed in light exposed control cells (black traces). Comparable results were obtained using another zebrafish cell line, AB-9, derived from adult zebrafish caudal fin (Fig. 1D,E) indicating that the H_2_O_2_ inducible expression of these genes is a general and not a cell type-specific property.

### Light and NOX-dependent ROS production regulates clock gene expression

**Figure 2:**
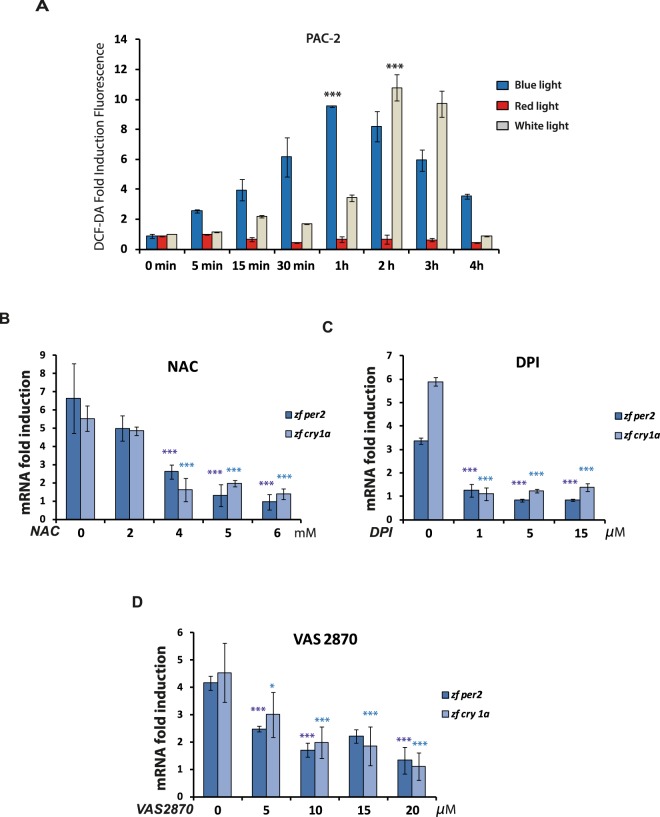
(**A**) Wavelength dependent ROS production. DCF-DA assay of PAC-2 cells exposed during 4 hours to different wavelengths of light. Blue, red and grey bars indicate ROS levels assayed upon blue, red and white light exposure, respectively. The means of fold induction (n = 24) ± SD with respect to time 0 are plotted on the y-axis, while the duration of light exposure is plotted on the x-axis. Levels of significance between peak points of DCF-DA fluorescence and time 0 are calculated by t-test and are indicated (***p < 0.001, **p < 0.01, *p < 0.05). (**B**–**D**) ROS-dependent, light-induced clock gene expression in PAC-2 cells. qRT-PCR analysis of expression of the light inducible genes *zfper2* (dark blue bars) and *zfcry1a* (light blue bars) in PAC-2 cells during blue light exposure in the presence of different concentrations of (**B**) NAC, (**C**) DPI and (**D**) VAS 2870. Samples were harvested after 3 hours of light exposure. On the y-axis is plotted the mean ± SD of mRNA fold induction with respect to the control samples incubated with the corresponding inhibitor-vehicle. The specific concentrations of NAC, DPI and VAS 2870 used are indicated on the x-axis of each graph. Levels of significance obtained in three independent experiments between the vehicle (0) and each inhibitor concentration are calculated by t-test and indicated (***p < 0.001, **p < 0.01, *p < 0.05).

We have previously shown that the induction of *zfper2* and *zfcry1a* occurs in a wavelength dependent manner, with blue light eliciting a stronger level of induction than red light^34^. Furthermore, it has been well established that illumination of cells with visible light triggers accumulation of intracellular ROS^30,35^. Thus, we wished to explore in more detail whether ROS may serve as a bridge between light and the acute induction of clock genes such as *zfper2* and *zfcry1a*. We first tested whether red and blue light can differentially induce intracellular ROS levels in our PAC-2 cells using a DCF-DA assay. An increase in ROS production in PAC-2 cells during 4 hours of white light or monochromatic blue-light (λ_peak_ = 468 nm) exposure (Fig. 2A, grey and blue bars, respectively) was observed. In contrast, upon exposure to a monochromatic red-light source (λ_peak_ = 657 nm) (Fig. 2A, red bars), no significant increase in ROS levels was observed for the entire duration of the experiment. Thus, light-induced ROS production in zebrafish cells appears to be wavelength dependent, with exposure to blue light being sufficient to significantly elevate intracellular ROS.

Next, we used a pharmacological approach to test the contribution of blue light-induced ROS levels to the induction of *zfper2* and *zfcry1a* expression. Specifically, we assayed the mRNA expression of these two clock genes triggered in PAC-2 cells by 3 hours of monochromatic blue light exposure, in the presence of three different ROS inhibitors: N-acetylcysteine (NAC), a general ROS scavenger (Fig. 2B); Diphenyleneiodonium (DPI), a general flavin-containing oxidase inhibitor (Fig. 2C); and VAS 2870, a well-validated NADPH oxidase inhibitor, which inhibits NADPH oxidase-mediated ROS production in cell free systems, cells and tissues, but which shows no intrinsic antioxidant activity and does not inhibit other flavoproteins^36^ (Fig. 2D). With all three inhibitors, we observed a significant reduction or a complete loss of blue light driven activation in *zfper2* and *zfcry1a* gene expression in a dose dependent manner. These results implicate NOX-generated ROS as playing a role in the activation of the two clock genes by blue light.

### The light responsive D-box enhancer element is a ROS target

**Figure 3:**
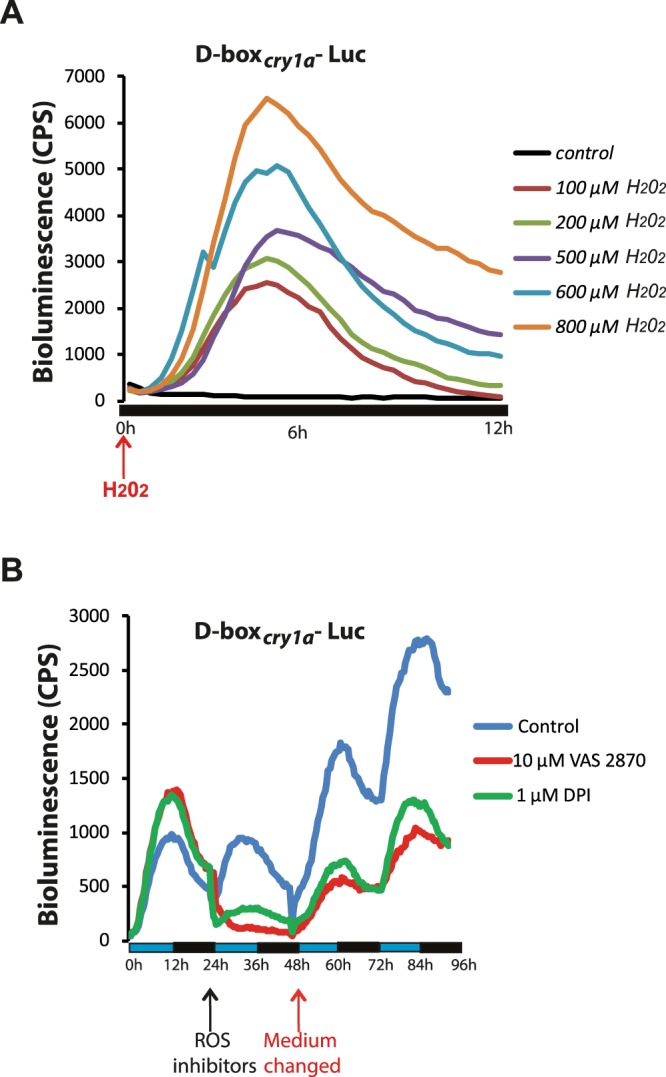
Impact of H_2_O_2_ and blue light on D-box enhancer-driven gene expression in PAC-2 cells. (**A**) Representative real time bioluminescence assay of PAC-2 cells transfected with the *D-box*_*cry1a*_*-Luc* reporter and treated with different concentrations of H_2_O_2_ (colour-coded traces). Black trace indicates cells treated with only the vehicle (control). (**B**) Representative real time bioluminescence assay of PAC-2 cells transfected with *D-box*_*cry1a*_*-Luc* and exposed to LD cycles without (control, blue trace) or with the ROS inhibitors DPI (green trace) and VAS2870 (red trace). Means of relative bioluminescence (n = 8) are plotted on the y-axis and time on the x-axis. Vertical arrows indicate times when the inhibitors were added (black arrow) or removed (red arrow). Blue and black bars below the graphic indicate the different lighting conditions during the experiment.

Previously, we have identified the D-box enhancer promoter element as being necessary and sufficient for light induced expression of *zfcry1a* and *zfper2*^26,27,37^ as well as other light inducible genes in zebrafish. Given that ROS production is responsible for triggering the *zfcry1a* and *zfper2* induction by light, we predicted it should also have an effect on the functionality of the D-box enhancer element. Thus, we tested whether H_2_O_2_ treatment (from 100 to 800 μM) of zebrafish cells was sufficient to activate bioluminescence from a luciferase reporter driven by a multimeric D-box enhancer sequence (*D-box*_*cry1a*_*Luc*^26^) in cells maintained under constant darkness. We observed a rapid increase followed by a progressive decrease in bioluminescence levels occurring during the first 12 hours in a H_2_O_2_ dose dependent manner (Fig. 3A). We confirmed that the observed increase of bioluminescence was not due to an artefact generated by the effect of H_2_O_2_ on luciferase enzyme activity by treating cells transfected with an SV40-driven luciferase reporter (pGL3 Control) with H_2_O_2_ or L15 medium (mock) (Fig. S2A). All these data reveal that the D-box enhancer serves not only as a light responsive element, but also acts to regulate transcription in a ROS dependent manner.

We next tested whether ROS inhibitors were also able to interfere with blue light induced, D-box directed gene expression. Specifically, PAC-2 cells were transfected with the *D-box*_*cry1a*_*Luc* reporter and exposed to blue light/dark cycles (LD). As expected, the cells showed a clear rhythm in bioluminescence with an increase observed immediately following “lights on” and a decrease after “lights off” during each cycle (Fig. 3B, left part of the panel). At the beginning of the second day, the cells were treated with VAS2870 (10 µM red line), DPI (1 µM green line), or vehicle as control (blue line) for 24 hours and then the inhibitors were removed from the cells for the remaining two LD cycles. During the ROS inhibitor incubation period (Fig. 3B, central part of the panel), the luciferase reporter activity was decreased significantly compared with the untreated control. Normal blue light-induced luciferase reporter activity was then restored after the inhibitors were washed out from the cells (Fig. 3B, right part of the panel). Together, these data point to the ROS pathway being at least in part required for the blue light-dependent induction of gene expression via D-box enhancer elements.

### Activation of stress-regulated MAPKs in PAC-2 cells in response to blue light

**Figure 4:**
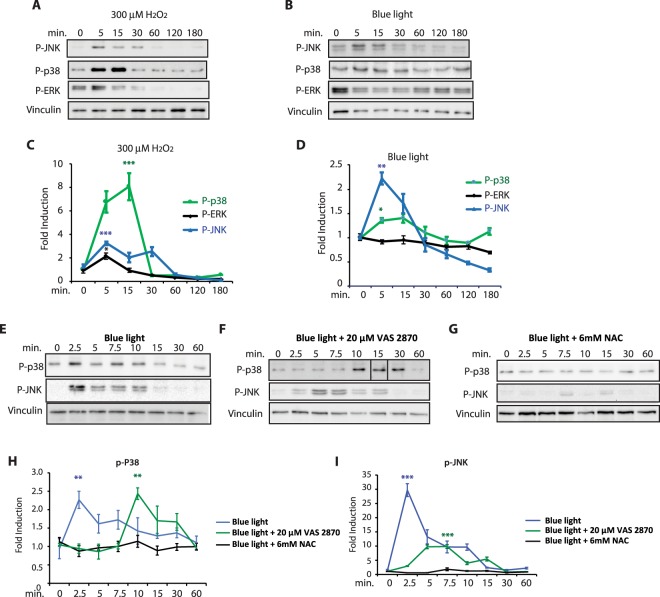
Activation of MAPKs by blue light and H_2_O_2_. (**A**,**B**) Western blot analysis and (**C**,**D**) its quantification of P-JNK, P-p38 and P-ERK levels in PAC-2 cells exposed either to blue light (**B**,**D**) or treated with 300 μM H_2_O_2_ (**A**,**C**) for 0, 5, 15, 30, 60, 120, 180 min. (**E–G**) P-JNK and P-p38 expression in PAC-2 cells pre-treated for two hours with (**E**) vehicle (control) (**F**) 20 μM VAS 2870, or (**G**) 6 mM NAC before blue light exposure. (**H**,**I**) Quantification of the western blotting data presented in (**E–G**). (**C**,**D**,**H**,**I**) Means of fold induction relative to time 0 ± SD are plotted on the y-axis and times are plotted on the x-axis. Levels of α-vinculin were used as a loading control. Quantification was performed with Image J software. Statistical analysis of the differences between time 0 and the peaks of expression is represented by asterisks (*) where *p < 0.05; **p < 0,01; ***p < 0,001. Each western blotting panel is assembled from cropped western blotting images (see Supplementary material file for the original images). In the case of the results for P-p38 in panel F, the 15 minutes sample has been digitally shifted to occupy the correct position in the chronology of the timecourse (as indicated in the original western blot image presented in the Supplementary material file).

**Figure 5:**
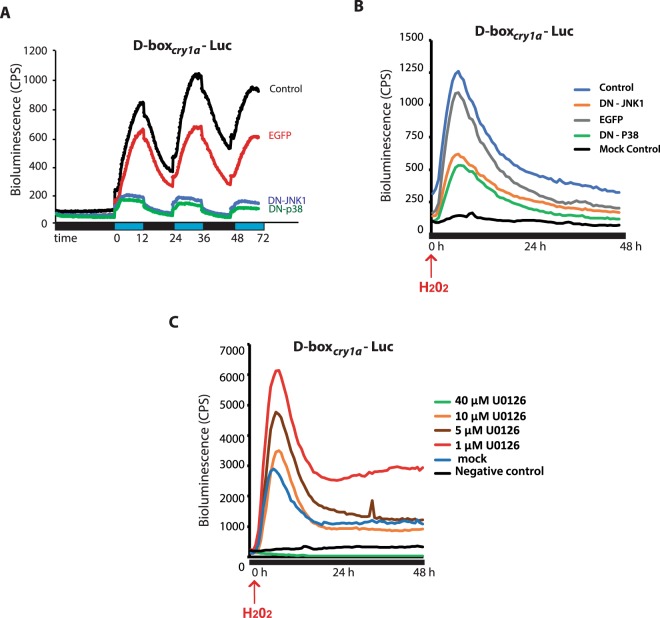
D-box regulation via MAP kinase signalling. (**A**,**B**) Representative real time luciferase assays of PAC-2 cells transfected with the *D-box*_*cry1a*_*-Luc* reporter together with expression vectors for DN-JNK1, DN-p38 or EGFP (negative control) upon exposure to (**A**) blue LD cycles or to (**B**) H_2_O_2_. (**C**) Representative real time luciferase assays of PAC-2 cells transfected with the *D-box*_*cry1a*_*-Luc* reporter and incubated with different concentrations of the U0126 inhibitor 1 hour before the H_2_O_2_ treatment. Mean of bioluminescence (CPS) (n = 8) are plotted on the y-axes and time is plotted on the x-axes. Blue and black bars below the x-axes denote the extent of the blue light and dark periods, respectively.

Which mechanism couples light-induced levels of ROS with the activation of D-box enhancer driven gene expression? It has been shown that ROS is able to activate a range of stress related signalling pathways. These include various MAP kinase pathways, notably p38, ERK and JNK. Furthermore, a previous study of the Z3 zebrafish cell line has reported inhibition of light-activated clock gene expression upon treatment with an ERK inhibitor^30,31^. However, using pharmacological and genetic approaches, our group has already revealed that the MEK/ERK MAP kinase pathway may serve as an inhibitor of blue light induced, D-box mediated gene expression in PAC-2 zebrafish cells^34^. On the basis of these previous data, we chose to explore whether blue light exposure and H_2_O_2_ can activate the other two stress related MAPK signalling elements, p38 and JNK. While in mammals, two JNK and eight p38 forms has been described^38^, in zebrafish, although there are numerous examples of gene duplication, a reduced number of MAPKs have been identified^39^. By western blot analysis using phospho-specific antibodies, we confirmed that the phosphorylated (activated) forms of zebrafish p38 (P-p38), and JNK (P-JNK) were induced by H_2_O_2_ treatment and importantly, also by blue light exposure (Fig. 4A–D). More specifically, we observed a transient induction of P-JNK levels after 5 minutes of H_2_O_2_ treatment followed by a rapid decrease (after 15 minutes) (Fig. 4A,C). In addition, a higher amplitude induction with similar kinetics was observed for P-p38 (Fig. 4A,C). The rapid induction of P-JNK and P-p38 levels in PAC-2 cells upon blue light exposure (Fig. 4B,D) was similar to that observed in the absence of light upon H_2_O_2_ treatment. In contrast, as we have previously described^34^, blue light failed to significantly change the ERKs phosphorylation state after 3 hours of blue light exposure (Fig. 4B,D black trace) compared to a transient, low amplitude induction observed 5 minutes following H_2_O_2_ treatment (Fig. 4A,C). Importantly, the activation by blue light observed in P-JNK and in P-p38 was attenuated by incubation of the cells with the two ROS inhibitors, VAS 2870 and NAC (Fig. 4E–I). Specifically, western blot analysis shows that addition of 6 mM NAC abolishes (Fig. 4G–I) and 20 μM VAS 2870 reduces and delays (Fig. 4F,H,I) the induction of P-JNK and P-p38 by blue light. To further confirm the role of P-JNK and P-p38 in the regulation of light- and H_2_O_2_-inducible gene expression, we used a genetic approach, by analyzing whether ectopic expression of dominant negative forms for JNK1 (dN-JNK1) and p38 (dN-p38) could affect the regulation of the *D-box*_*cry1a*_*Luc* reporter by blue light and H_2_O_2_ in PAC-2 cells (Fig. 5A,B). We observed a significant reduction of light- and H_2_O_2_-dependent D-box driven activation upon co-expression of dN-JNK1 or dN-p38, supporting a role for both kinases in light- and H_2_O_2_-regulated gene expression. In contrast, no significant reduction in D-box activation was observed in PAC-2 cells ectopically overexpressing a control EGFP expression construct. In addition, we used a pharmacological approach to test the involvement of ERK signalling in the response of the D-box to ROS. H_2_O_2_-treated PAC-2 cells were exposed to various concentrations of the ERK inhibitor, U0126 (Fig. 5C). As we previously reported for photic induction of the D-box^34^, our results revealed that lower U0126 concentrations (1 to 5μM) significantly enhanced the D-box activation by H_2_O_2_, consistent with a general inhibitory effect of the ERK pathway on D-box enhancer function. In contrast, the highest concentration of U0126 (40 μM) completely abolished ROS-induced D-box activation consistent with previous reports of a non-specific effect of this high concentration of inhibitor^40^.

### Evolution of the Light/ROS-dependent signalling pathway

Given the absence of direct light sensing peripheral clocks in higher vertebrates such as mammals, a key question is how has this ROS-dependent, light input pathway adapted during evolution. To tackle this issue we have examined the behaviour of key elements in this pathway in two distinct settings. Firstly in the context of mammalian cell lines and secondly during evolution under extreme environmental conditions, in the Somalian blind cavefish *Phreatichthys andruzzii*.

**Figure 6:**
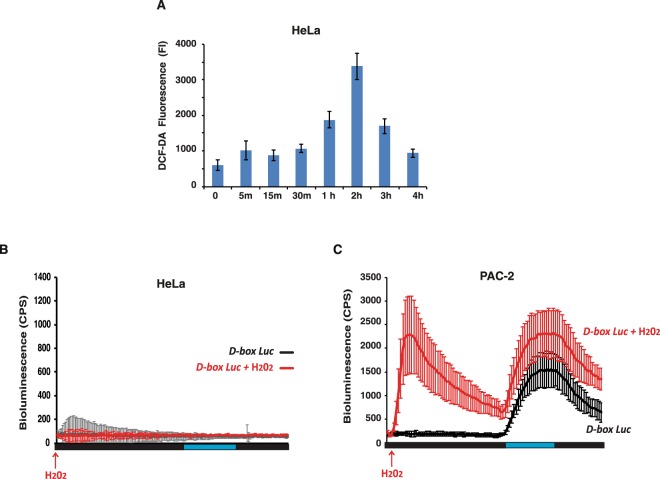
ROS and D-box regulation in HeLa cells (**A**) DCF assay of HeLa cells exposed during 4 hours to monochromatic blue light. Means of fold induction (n = 24) ± SD with respect to time 0 are plotted on the y-axis, while the duration of light exposure is plotted on the x-axis. (**B**,**C**) Representative real time bioluminescence assays in **(B**) HeLa and (**C**) PAC-2 cells (control) transfected with the D box-driven-luciferase reporter *D-box*_*cry1a*_*-Luc*, treated with H_2_O_2_ and after 24 hours exposed to one blue light/dark cycle (*D-box Luc* + H_2_O_2_, red traces). Black traces indicate cells treated with only the vehicle and after 24 hours exposed to one blue light/dark cycle (*D-box Luc*). Means of relative bioluminescence (n = 8) are plotted on the y-axis and time on the x-axis. Blue and black bars below the graphics indicate the different lighting regimes during the experiments. See also Fig. S2 for controls.

Similar to the results observed in zebrafish cells (see Fig. 2A) and consistent with previous studies^41^, blue light exposure induces ROS production in mammalian cells (Fig. 6A). Does light-induced ROS influence the D-box enhancer element in mammalian cells? The role of the D-box in the circadian clock is fundamentally different between fish and mammals. In mammals, where cells are not directly light responsive, the D-box is a clock output element^42^ driving rhythmic gene expression under clock regulation. Instead, in fish this enhancer plays a role in the clock input pathway^26,27,37^ responding directly to light and driving expression of genes that in turn are able to entrain the circadian clock. Our bioluminescence assays of HeLa cells, transfected with our *D-box*_*cry1a*_*-Luc* construct and treated with 300 μM H_2_O_2_ or blue light (Fig. 6B), showed no significant induction in D-box driven bioluminescence in the mammalian cells compared to zebrafish cell controls (Fig. 6C). As a positive control for the functionality of the D-box enhancer element reporter in HeLa cells, co-expression with one of the D-box binding transcription factors, TEF1 (Thyrotroph embryonic factor 1)^37,43,44^ resulted in strong reporter gene activation (Fig. S2B). Thus, in mammals the D-box does not seem to respond directly to ROS signalling or blue light exposure.

**Figure 7:**
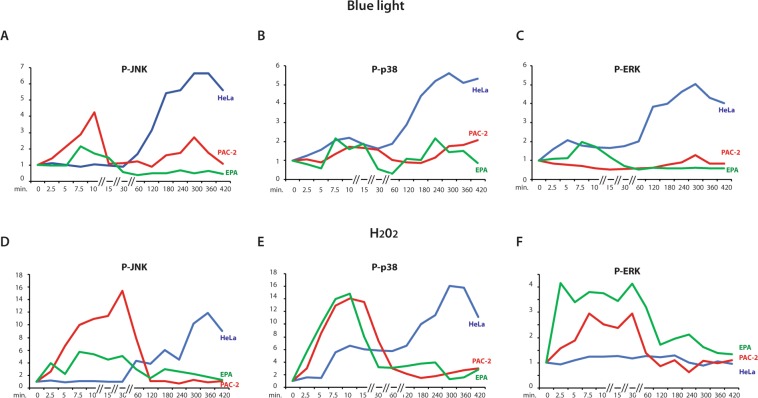
Regulation by light and ROS of MAP kinases. (**A–F**) Western blot quantification of PAC-2 (red traces), HeLa (blue traces) and EPA (green traces) cells treated for 420 minutes (7 hours) with (**A–C**) monochromatic blue light or (**D**,**E**) 300 μM H_2_O_2_ for (**A**,**D**) P-JNK, (**B**,**E**) P-p38 and (**C**,**F**) P-ERK levels. Mean values (n = 2–3) are plotted on the y-axis and time on the x-axis. Quantification was performed using Image J software and the values were normalized for the expression of vinculin. See Fig. S3 for representative western blotting data and Supplementary material file for the original images.

We then explored the regulation of the stress-activated MAP kinase signalling pathway in HeLa cells in response to blue light. Interestingly, we observed a predominantly delayed and sustained response (from 2 to 7 hours) in the induction of P-p38, P-JNK and also P-ERK (Fig. 7A–C, blue traces and Fig. S3). This contrasts with the rapid and transient response of P-p38, P-JNK and the lack of a response for P-ERK observed in the PAC-2 cells during the first 30 minutes of blue light exposure (Fig. 7A–C, red traces and Fig. S3). Interestingly, analyzing PAC-2 cells with the same 7 hours time course of blue light exposure, we also observed a second delayed induction of all three kinases occurring after 4 hours. The delayed peak of activation in PAC-2 cells was similar in timing to the main peak observed in mammalian cells. Upon H_2_O_2_ treatment in HeLa cells, a delayed sustained induction was detected only for P-p38 and P-JNK (Fig. 7D–F, blue traces and Fig. S3) compared with the early, transient induction observed in zebrafish cells (Fig. 7D–F red traces, Figs 4 and S3). Thus, these results point to major differences between fish and mammalian cells in terms of the timing of the MAP kinase response to light as well as ROS. In addition, these data show that the D-box enhancer element is not a direct ROS target in this human cell line. This is consistent with a fundamental shift in the role of the D-box enhancer element during vertebrate evolution.

**Figure 8:**
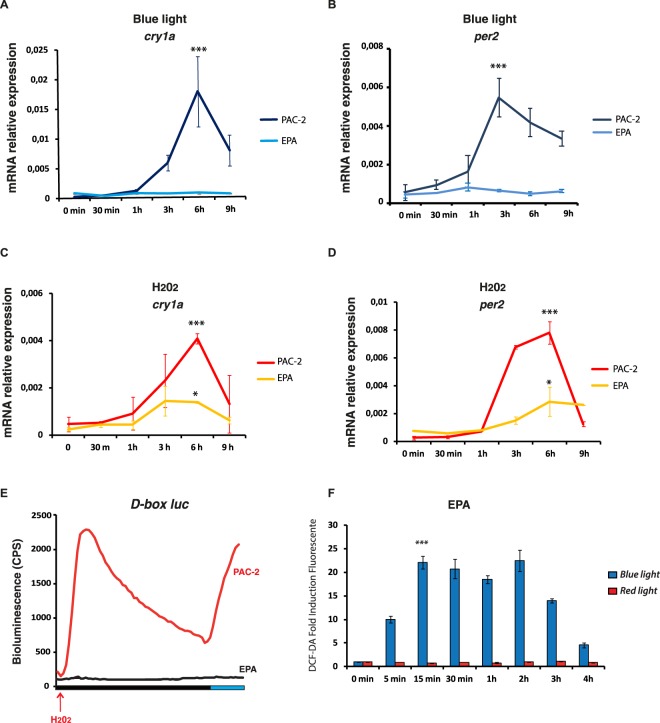
Expression of clock genes and the D-box reporter construct in cavefish and zebrafish cells. qRT-PCR analysis of (**A**,**C**) *cry1a* and (**B**,**D**) *per2* in cells exposed for 9 hours to (**A**,**B**) blue light and (**C**,**D**) 300 μM H_2_O_2_. Mean mRNA relative expression (n = 3) ± SD is plotted on the y-axis, whereas time is plotted on the x-axis. Statistical tests of the differences in kinetics of gene expression between the two cell lines were analyzed with two-way ANOVA followed by Bonferroni multiple comparable T-test. Statistically significant differences between peaks of expression are indicated (*p < 0.05; **p < 0,01; ***p < 0,001). (**E**) Representative real time luciferase assay of PAC-2 (red trace) and EPA cells (black trace) transfected with the *D-box*_*cry1a*_*-Luc* reporter and treated with H_2_O_2_ and blue light (left and right sides of the panel, respectively). Means of bioluminescence (CPS) (n = 8 wells) are plotted on the y-axis and the extent of the dark and blue light periods are indicated on the x-axis. See Fig. S2B for controls. (**F**) DCF-DA assay of EPA cells during 4 hours of exposure to blue (blue bars) or red (red bars) light. The mean of fold induction ± SD with respect to time 0 (n = 24) are plotted on the y-axis and time on the x axis. Levels of significance between peak points of expression are indicated (***p < 0.001, **p < 0.01, *p < 0.05).

We next explored how evolution under extreme photic conditions affected the light/ROS dependent signalling pathway. We have already shown that the cavefish *P. andruzzii* displays a natural loss of function for light-induced gene expression^28^. For this study, we established an embryonic cavefish *P. andruzzii* cell line, EPA (**E**mbryonic **P**. **A**ndruzzii), comparable with the zebrafish PAC-2 line. Specifically, both lines were derived from dissociated embryos of comparable developmental stages (36 hpf for PAC-2 and 26 hpf for EPA^45,46^). As expected, in the EPA cell line, neither the clock genes *cfper2* and *cfcry1a* (Fig. 8A,B) nor a D-box-driven luciferase reporter (Fig. 8E, black trace, right side of panel) were induced following blue light exposure confirming the lack of light responsiveness in this cavefish *in vitro* model. However, as previously observed for the PAC-2 and HeLa cells, blue light exposure of the EPA cells does result in an increase in intracellular ROS levels (Fig. 8F). Treatment of EPA cells with H_2_O_2_ was able to induce *cfper2* and *cfcry1a* expression, although with a significant reduction in amplitude compared with that observed in the PAC-2 cells (Fig. 8C,D). Importantly, as in the case of mammalian cells, acute treatment of EPA cells with H_2_O_2_ failed to activate D box-driven luciferase expression (Fig. 8E, black trace left side of the panel). As a positive control for the functionality of the D-box enhancer element reporter in EPA cells, co-expression with TEF1 resulted in strong reporter gene activation (Fig. S2B). Together, our results point to cavefish cells retaining the partial ability to upregulate clock gene expression by ROS via a D-box independent mechanism.

Finally, we tested the activation of the stress-regulated MAP kinases in EPA cells by blue light, as well as H_2_O_2_ treatment. Cavefish cells showed a rapid transient induction of P-JNK P-p38 and P-ERK (Fig. 7 green traces) for both the treatments. The blue light-induced P-ERK levels observed in EPA cells (Fig. 7C green trace) contrasts with the relatively stable levels documented in zebrafish cells.

These data reveal that during vertebrate evolution, major changes in the responsiveness of peripheral clocks to light have been accompanied not only by changes in the photoreceptor repertoire, but also by significant alterations in ROS-responsive signal transduction pathways.

## Discussion

The data presented in this study point to a central role for ROS in the clock’s light input pathway in fish cells. We have demonstrated that blue light exposure triggers a rapid increase in intracellular ROS, involving the activity of flavin-containing oxidases, specifically NADPH oxidase. ROS in turn is able to activate two stress-associated MAP kinases, p38 and JNK. This final step appears to induce the expression of the light-inducible clock genes *cry1a* and *per2* by transcriptional activation via D-box enhancer elements in their promoters. Following induction of *cry1a* and *per2* expression, the phase of the circadian clock is adjusted to match the lighting conditions.

### ROS as a signalling molecule

While ROS can generate cellular damage by reacting with DNA, proteins and lipids, it is increasingly clear that it can also act as an internal signalling molecule. Our findings that ROS serves as a key circadian clock regulator, are consistent with several previous reports from a range of different model systems indicating close links between clocks and oxidative stress^30,47–51^. Our findings that predominantly blue light triggers ROS accumulation in fish cell lines, is also consistent with the ability of violet/blue light to initiate the photoreduction of flavin which can then activate flavin-containing oxidases^35,52^. We reveal a general role for NADPH-flavin-containing oxidases (NOXes) in the regulation of light inducible gene expression using DPI, a general Flavin inhibitor and VAS2870, a well validated NOX inhibitor (non isoform-specific)^36^. These two inhibitors were able to block JNK and p38 phosphorylation, D-box driven transcriptional activation as well as induced *cry1a* and *per2* gene expression in cells upon blue light exposure. In eukaryotes, flavin containing NOXes represent one of the key sources of cellular H_2_O_2_^53^. However, the mechanisms responsible for activation of NADPH oxidases are still incompletely understood. In some cases, protein kinase C (PKC) activation has been implicated as a critical step triggering phosphorylation of cytoplasmic subunits of the NADPH oxidase complexes (p47 phox) with subsequent enzyme assembly. In other cases, the NOX enzyme complex appears to be directly and reversibly regulated by Ca^2+^ levels^53–55^. The work presented here supports a direct effect of blue light on the activation of NADPH oxidases via flavin photosensitivity playing a central role in the light input pathway in fish.

### Additional light-regulated signalling pathways

Does ROS activated signalling represent the only pathway relaying light to changes in clock gene expression? Our results indicate that there are additional signalling elements in the light input pathway. For example, red light exposure also induces *cry1a* and *per2* gene expression in zebrafish cells via the D-box enhancer element although with a lower amplitude^28^. However, red light does not trigger significant ROS production. Importantly, we have also demonstrated that opsins play a key role in light regulated circadian clock gene expression^28^. Specifically, *P. andruzzii* carries loss of function mutations in Melanopsin (Opn4m2) and TMT-opsin (TMT) which at least in part contribute to the blind peripheral clock phenotype. Thus, a key question is what is the relative contribution of opsins and flavin-containing oxidases to the regulation of the light input pathway? One potential mechanism could involve activated opsins signalling through a ROS-independent pathway. However, at the level of the signalling target, i.e. D-box binding transcription factors, this signal would require convergence with a ROS–derived signal in order to trigger gene activation. In an alternative mechanism, the opsins would be coupled via G-proteins to the flavin - NADPH oxidase. Thereby, opsins activation by light could directly trigger ROS production by NADPH oxidase for intracellular signalling. In support of this model, previous studies have confirmed that G-protein-coupled receptors are indeed able to directly activate NADPH oxidases^56,57^. In zebrafish, the existence of 42 different opsins, many of which are expressed in peripheral tissues, implies a degree of redundancy in opsin photoreceptor function in fish cells^29^. Therefore, it appears likely that there is inherent complexity to light responsive signalling pathways in peripheral tissues.

### Light-dependent function of stress-activated MAP Kinases

In contrast to our previous identification of ERK as a negative regulator of light induced gene expression^34^, our current data implicate the stress-activated MAP kinases p38 and JNK as key positive signalling elements linking light exposure and the induction of clock gene expression. Our findings predict that the PAR/E4BP4 family of transcription factors, such as DBP, TEF and HLF, that have been shown to bind to and regulate the D-box enhancer element^43,44^, could play a crucial role as regulatory targets of these stress-activated kinases.

However, these evolutionary conserved kinases also exhibit pleiotropic functions related to the control of cell growth, differentiation, development, cell cycle, survival and cell death. A key intermediate signal in the photic response pathway appears to be ROS. Indeed, it has been well documented that oxidative stress represents one of the key activators of the p38 and JNK stress-activated MAP kinases, and is predicted to act via oxidative modification of the kinase proteins themselves or possibly by inactivation of MAPK phosphatases (MKPs)^58^. Therefore, one important question is how do cells differentially interpret a light-derived ROS signal destined to specifically regulate the phase of the clock from a more general oxidative stress event that might merit a more global cellular response. In this regard, our results have revealed complexity in the kinetics of activation of these light and ROS regulated kinases. Specifically, exposure of zebrafish cells to light results in a bimodal induction of P-JNK and P-p38, with a very rapid transient induction occurring within the first 10 minutes of illumination, followed by a slower, delayed activation occurring up to 2–3 hours after initial light exposure. Treatment with H_2_O_2_ leads to the early, rapid and transient induction of the stress-activated kinases but fails to elicit the second, delayed induction event observed upon prolonged illumination. Furthermore, our assays of ROS levels in cells exposed to light have documented significant increases in ROS levels occurring within 5 minutes of light exposure while peak levels of ROS are achieved only after 1–2 hours.

Given these results, it is tempting to speculate that in zebrafish cells, following light exposure or treatment with H_2_O_2_, crossing a critical low threshold of accumulating ROS levels leads to a transient activation of p38 and JNK, possibly then downregulated via the activation of MKPs. This early event is “interpreted” as a photic signal and so ultimately leads to activated clock gene expression. Subsequently, continued exposure to light and sustained accumulation of ROS above a much higher threshold, results in a second wave of kinase induction that may constitute a signal for a more general cellular stress response. Indeed, in this regard, it has been well documented that prolonged exposure to blue light can trigger cytotoxic effects^41^.

### The evolution of light input pathways

**Figure 9:**
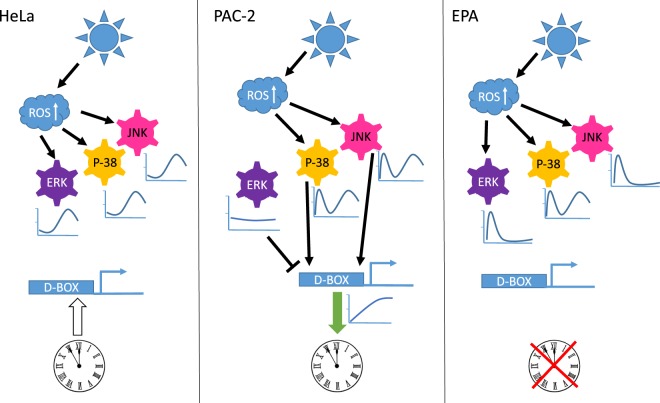
Light-driven signalling to the circadian clock via ROS, the MAPK cascade and the D-box enhancer in vertebrate cells. Schematic representation of how exposure to blue light differentially influences MAPK signalling and D-box enhancer-driven gene expression. In all three cell lines studied, blue light exposure triggers an increase in intracellular ROS levels. In PAC-2 cells (central panel), this results in two peaks of activation of p38 and JNK, one rapid (5–15 mins) and a second delayed increase (6–7 hours). In contrast, levels of P-ERK remain relatively unchanged during light exposure. This combined signalling results in the activation of D-box-driven gene expression, ultimately leading to circadian clock entrainment (indicated by green arrow). In HeLa cells (left panel), all three MAP kinases are activated with a predominantly delayed response (6–7 hours) that does not influence D-box driven transcription. Indeed, in mammalian cells D-box regulated expression constitutes a clock output pathway (indicated by white arrow). In EPA cells (right panel), all three MAP kinases are activated rapidly and transiently (with p38 also exhibiting the second, delayed peak of activation). However, as for the HeLa cells, this signalling does not affect the D-box enhancer or entrain the circadian clock. Indeed, it has been previously shown^28^ that cavefish cells possess a blind circadian clock (indicated by a red cross)

Our comparison of light triggered signalling events between zebrafish cells, blind cavefish and mammalian cells have provided important new insight into the normal function of these pathways as well as contributing to an understanding of how signal transduction pathways have adapted during evolution (Fig. 9).

In blind cavefish, during evolution in a perpetually dark, cave environment, the normal peripheral light sensing mechanisms have changed considerably^59^. Thus, while light exposure still triggers an increase in intracellular ROS as well as a rapid, transient induction in all three MAP kinases, only P-p38 levels exhibit the second, delayed increase observed in zebrafish cells. In contrast, H_2_O_2_ treatment triggers rapid increases in the levels of activation for all three kinases in a similar fashion to those described in zebrafish. However, despite the conservation of many aspects of these early signaling events, neither ROS nor light exposure is able to trigger activation of transcription via the D-box enhancer element. This predicts that in cavefish, loss of function mutations affect elements which bridge the stress-activated kinases and the regulation of transcription at the D-box enhancer. In this regard, it is interesting to note that the tonic activity of the ERK pathway upon blue light exposure in zebrafish, contrasts with the activation of this same pathway in the cavefish cells. Therefore, in zebrafish, ERK activity might serve as a tonic negative regulatory signal for the PAR/E4BP4 transcription factor family^34^ and the balance between ERK, JNK and p38 activity defines the kinetics of D-box driven gene expression. However, in the case of cavefish, light exposure results in activation of the ERK pathway, with consequently, a potential block of the gene expression response to light via PAR/E4BP4 driven D-box activation.

As for the blind cavefish *P. andruzzii*, mammalian cells also fail to exhibit light induced D-box driven clock gene expression. Furthermore, in mammals the D-box enhancer occupies a completely different role within the circadian clock mechanism, serving as a clock output target^42^. Here, consistent with previous reports^35^, we reveal that light exposure does trigger accumulation of ROS in the HeLa cell line in a similar fashion to that described in the fish cell lines. However subsequently, unlike the fish species, a predominantly delayed activation of all three MAP kinases is observed. In addition, upon H_2_O_2_ treatment, again, a delayed induction of P-JNK and P-p38 is evident. Furthermore, neither light nor H_2_O_2_ subsequently triggers D-box driven transcription. Together, these results suggest that the increase in ROS levels observed in these mammalian cells represent a delayed stress response and play no role in relaying light information to the clock.

Our data comparing mammalian and cavefish cells, illustrate that far from being static and highly constrained, there is significant plasticity in the function and regulation of light/ROS responsive signalling pathways during evolution. The regulation of the stress-activated MAP kinases and in particular the function of the D-box exhibits considerable differences between different vertebrate groups. In the case of the blind cavefish, loss of specific opsin photoreceptors, as well as ROS and light-responsive D-box enhancer function is associated with evolution under extreme photic conditions. In the case of mammals, there has been speculation that the loss of peripheral clock photoentrainment is linked with a “nocturnal bottleneck” event during early mammalian evolution^60,61^. At a time when the mammalian ancestors competed with diurnal dinosaurs, they are predicted to have adopted an exclusively nocturnal, subterranean existence to avoid predation. It has been speculated that this lifestyle may have resulted in major changes in the organization of the circadian timing system with the centralization of photic input to the retina, reduction in the complexity of the opsin gene repertoire and the establishment of the SCN as a specialized central coordinating pacemaker for the multiple peripheral clocks^62^. Therefore, it is tempting to speculate that as in blind cavefish, the observed changes in D-box and MAP kinase responsiveness to ROS in mammals might reflect a fundamental facet of evolution under extreme photic conditions.

## Materials and Methods

### Ethics statements

All husbandry and experimental procedures were performed in accordance with European Legislation for the Protection of Animals used for Scientific Purposes (Directive 2010/63/EU), the German (Animal Protection Law, BGBl. I, 1934, 2010) and Italian (D.lgs. 26/2014) animal protection standards. Research was also approved by the Local Government of Baden-Württemberg, Karlsruhe, Germany (Az.: 35-9185.81/G-130/12), and by the University of Ferrara Institutional Animal Care and Use Committee and the Italian Ministry of Health (auth. num. 890/2016-PR). General license for fish maintenance and breeding: Az.: 35-9185.64 for the Karlsruhe Institute of Technology, and 47/2013-A for the University of Ferrara.

### Establishment of the *P. andruzzii* cavefish embryonic cell line (EPA)

After hormonal induction of reproduction in adult *P. andruzzii* by intraperitoneal injection of LRH (Sigma Aldrich 0,05mg/g body weight) and Pimozide (Sigma Aldrich 2,5 μg/g body weight), fertilized eggs were cleaned with sterile E3 medium (5 mM NaCl, 0.17 mM KCl, 0.33 mM CaCl_2_, 0.33 mM MgSO_4_) in the presence of 10^−5^% Methylene Blue. At 6 hpf, eggs were incubated for 5 minutes in E3 plus 30 μg/ml of Pronase (Roche) to soften the chorion and immediately washed 3 times with PBS 1X. Embryos were then left to develop at 26 °C until 26 hpf, when the embryos were trypsinized (Gibco BRL) for 5 minutes and then dissociated tissues were plated in a cell culture flask (Greiner) in L15 (Leibovitz) culture medium (Gibco BRL) supplemented with 20% Fetal Calf Serum (Sigma Aldrich), 2% Penicillin/Streptomycin and 0.2% fungicide (Gentamicin, Gibco BRL 50 mg/ml stock). Established EPA cells were then maintained as described below.

### Zebrafish, cavefish and mammalian cell culture maintenance

The zebrafish PAC-2^63^ and cavefish EPA embryonic cell lines as well as the zebrafish adult cell line AB-9^64^ were propagated at 26 °C in L-15 (Leibovitz) medium (Gibco BRL) supplemented with 15% or 20% (cavefish) Fetal Calf Serum (Sigma Aldrich). The medium was supplemented with 100 units/ml penicillin, 100 µg/ml streptomycin and 50 µg/ml gentamicin (Gibco BRL) in an atmospheric CO_2_, non-humidified cell culture incubator. The HeLa (human cervical cancer derived) cell line was cultured at 37 °C with 5% CO_2_ in Dulbecco’s Modified Eagle’s Medium (DMEM) containing 10% heat inactivated fetal calf serum (FCS), 2 mM L-glutamine, 50 µg/ml penicillin and 50 µg/ml streptomycin. For cell maintenance, confluent cultures were routinely split after trypsinization with 0.25% (w/v) Trypsin.

### Hydrogen Peroxide and Pharmacological treatments

Hydrogen Peroxide treatments were performed by diluting a 1M stock solution (Sigma Aldrich, H1009) in L15 medium at a final concentration between 100–800 µM depending on the experiment. Treatments with VAS2870, DPI, N-acetylcysteine and U0126 were all performed as recommended by the manufacturers and according to the literature. Details of the range of stock concentrations, final concentrations and dilution medium for each compound are indicated in Table S1. In the respective results sections and figure legends, the specific concentrations used for each experiment are also indicated.

### Phase Response Curve Analysis

Zebrafish PAC-2 cells stably expressing the *zf per1b-Luc* reporter^25^ were plated in 96 well plates in medium supplemented with luciferin. Plates were exposed for 3 days to an LD cycle. On the fourth day, cells were transferred to constant darkness. Then, individual sets of wells on the plate were treated with 300 μM of H_2_O_2_ at different times during the subjective day and night. Then bioluminescence was assayed for the following days in DD. Phase shifts were then calculated relative to the DD control on the third day. The time of delivery of each H_2_O_2_ treatment was expressed in circadian time (CT), where CT0 is defined as the beginning of the subjective day and CT12, the beginning of the subjective night.

### Light sources

Cell illumination was performed at a constant temperature using one of the following LED light sources adjusted to deliver the same photon flux (1.42 × 10^18^ ± 0.04 × 10^18^ photons/s/m^2^): White light-emitting diodes (LEDs, Kopa 440 nm-690 nm); monochromatic blue (λ_peak_ = 468 nm, Kopa) and red LEDs (λ_peak_ = 657 nm, Kopa).

### Measurement of intracellular ROS

The level of intracellular ROS was detected using 2′,7′-dichlorofluorescein diacetate (DCFH-DA) (Sigma D6883-50MG) according to the manufacturer’s instructions. Specifically, 3 × 10^4^ cells were seeded per well in a 96-well plate (CELLSTAR, Greiner Bio-One) and incubated for 24 hours at 25 °C in the dark. Then cells were exposed to the different light sources (LEDs) or remained in darkness as a control, for a 4 hours period. At different time points the cells were assayed after 30 min of incubation with 200 µl of 10 µM DCFH-DA in L15 medium (lacking phenol red) at 26 °C or at 37 °C for fish or mammalian cells, respectively. After one wash with ice-cold PBS, the cell fluorescence produced by the oxidation via ROS of DCFH-DA to DCF (2′, 7′- dichlorofluorescein) was measured with a FluoStar Optima fluorescent chemiluminescence analyzer at 490 nm excitation and 530 nm emission wavelengths.

### Quantitative RT-PCRs

Total RNA was extracted with Trizol Reagent (Gibco, BRL) according to the manufacturer’s instructions. Reverse transcription was performed using Superscript III RT (Invitrogen). A StepOnePlus Real-Time qRT-PCR System (Applied Biosystems) and SYBR Green I fluorescent dye (Promega) were used. Expression levels were normalized using *β-actin*. The relative levels of mRNA were calculated using the 2^−ΔΔCT^ method. For each gene, primer sequences are presented in Table S2.

### Protein analysis and Western Blotting

Total protein extracts were prepared by the direct addition of 1X Laemmli buffer (6% SDS, 20% glycerol, 125 mM Tris pH6.8, 0.01% bromophenol blue, 100 mM DTT) including a 1x cocktail of protease and phosphatase inhibitors (Sigma Aldrich P5726 and P8340, respectively). Gel electrophoresis was performed in a SDS polyacrylamide gel in a Biorad miniprotean system 3 chamber and transferred to a Hybond-P membrane (Millipore) by electro-blotting (Biorad). Binding of each antibody was visualized using the ECL detection system (Biorad). All images were acquired and analyzed using Image Lab™ Software (BioRad, USA). Images were then quantified using Scion Image software (NIH, http://rsb.info.nih.gov/nihimage/). Finally, for the preparation of figures, these original images were digitally cropped (see Supplementary material file for original image data). The antibody concentrations used were selected according to the manufacturers’ recommendations. All the antibodies used are listed in Table S3.

### Luciferase assays

Cell transfections were all performed using FuGene HD (Promega) or ScreenFect (S-4001 InCella) reagents according to the manufacturers’ protocols. The real-time bioluminescence assays were performed and analyzed as described previously^65^. Bioluminescence was assayed with a Topcount NXT automatic scintillation counter (Perkin Elmer). Data were imported into Microsoft Excel using the “Import and Analysis” macro (S. Kay, Scripps Research Institute). The *in vitro* luciferase assays were performed using the Luciferase Assay System kit (Promega) according to the manufacturer’s instructions. Cells were transfected with 250 ng of reporter plasmid together with 50 ng of the β-galactosidase expression construct to control for transfection efficiency and 1 ng or 5 ng of the transcriptional activator TEF1 expression construct. Luciferase activity was measured using a Victor Multilabel Plate Reader (Perkin Elmer). A list of the luciferase reporters and transcription factor expression constructs are reported in Table S4 including the expression vectors for the dominant negative p38 (DN-p38, *pcDNA-3 Flag p38 alpha (agf)*^66^) and dominant negative JNK1 (DN-JNK1, *pcDNA-3 Flag Jnk1a1(apf)*^67^) purchased from Addgene (#20352 and #13846, respectively).

### Statistical analysis

Student’s t-test analysis was performed using Microsoft Excel software. One-way or two-way analysis of variance (ANOVA) followed by Bonferroni’s multiple comparison tests were performed using GraphPad Prism 4.0 (http://www.graphpad.com).

All the results are expressed as means ± SD of at least three biological replicates. In the statistical tests p < 0.05 was considered statistically significant. In each figure, p < 0.05, p < 0.001 and p < 0.0001 are represented by *, ** and *** respectively.

## Electronic supplementary material

Supplementary Figures and Tables

Original western blot images
