# Supplementary material for: Evolution shapes the responsiveness of the D-box enhancer element to light and reactive oxygen species in vertebrates
Source: Sci Rep. 2018 Sep 4;8:13180. doi: 10.1038/s41598-018-31570-8 (PMC6123470; doi:10.1038/s41598-018-31570-8)
Supplement: Supplementary file 1 — Supplementary Figures and Tables [file 41598_2018_31570_MOESM1_ESM.pdf]

# Evolution shapes the responsiveness of the D-box enhancer element to light and reactive oxygen species in vertebrates.

Cristina Pagano, Rima Siauciunaite, Maria L. Idda, Gennaro Ruggiero, Rosa M. Ceinos, Martina Pagano, Elena Frigato, Cristiano Bertolucci, Nicholas S. Foulkes and Daniela Vallone

## Supplementary material

### Figure S1

A

B

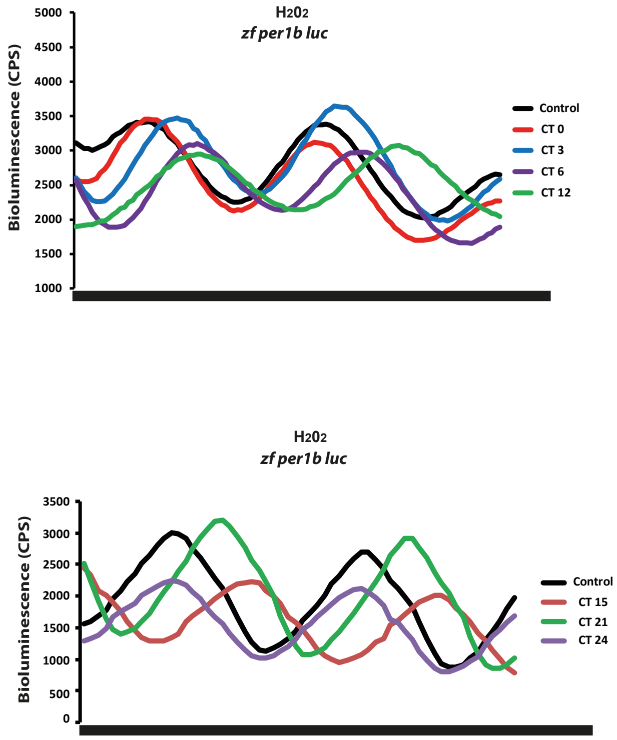


**Figure S1: $H_2O_2$ entrains the zebrafish cell circadian clock. (A, B)** Luciferase expression profile of the *zf per1b-luc* luciferase construct stably expressed in PAC-2 cells upon $H_2O_2$ treatment delivered at different time points during the subjective **(A)** day and **(B)** night (CT). Relative bioluminescence is plotted on the y-axis and time on the x-axis. Each trace is plotted as the mean from a minimum of n=4 independent wells.

## Figure S2

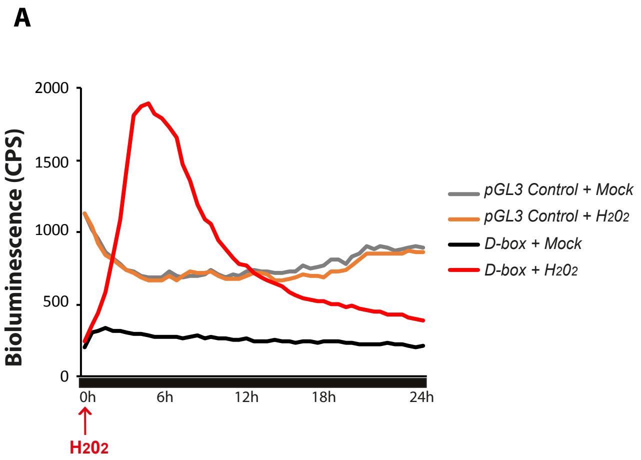


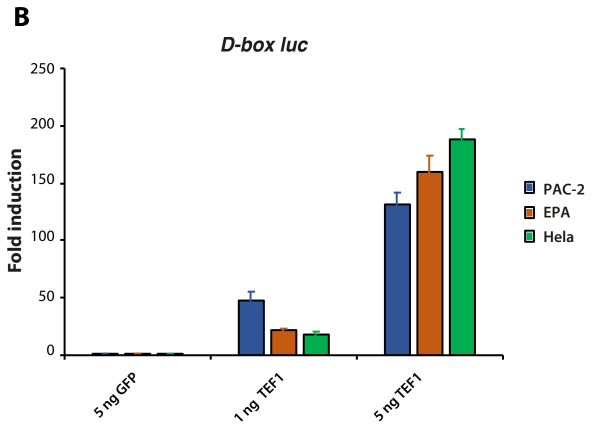


**Figure S2: D-box activation by $H_2O_2$ . (A)** Real time luciferase expression profile of D-box and SV40-driven (*pGL3 Control*) luciferase reporters in PAC-2 cells upon treatment with 300μM $H_2O_2$, during a 24 hours period. As a negative control for the $H_2O_2$ treatment, PAC-2 cells were mock treated with L15 medium. The black bar below the panel indicates the constant darkness during the experimental procedure. Relative bioluminescence is plotted on the y-axis and time on the x-axis. Each trace is plotted as a mean of n=4 independent wells. **(B)** *in vitro* luciferase assay of PAC-2, EPA and HeLa cells co-transfected with the *D-box Luc* reporter and two concentrations (1ng and 5ng) of the expression vectors for the TEF1 transcription factor or EGFP (negative control). Mean (n=3) ± SD of the relative fold induction of bioluminescence relative to the control is plotted on the y-axis and the expression vector with its concentration is indicated on the x-axis.

## Figure S3

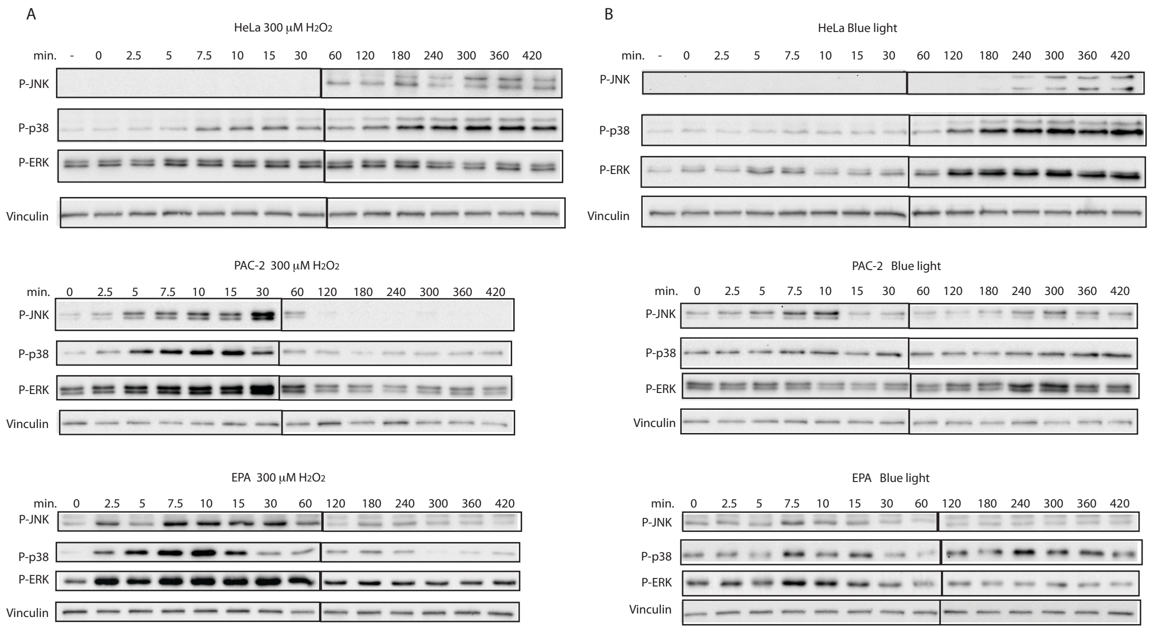


**Figure S3: Regulation by light and ROS of stress-induced MAP kinases. (A, B)** Representative western blots for P-JNK, P-p38, P-ERK and vinculin as a loading control in HeLa (upper panels), PAC-2 (central panels), and EPA (lower panels), treated for 420 minutes (7 hours) with **(A)** 300 μM $H_2O_2$ or **(B)** blue light. Each panel is assembled from cropped western blotting images (see Supplementary material file for the original images). Specifically, for all PAC-2 and HeLa data, each time course is assembled from two identically and simultaneously processed western blots, where the first blot contained samples from time 0 to 30 minutes of treatment and the second contained samples from 60 to 420 minutes. In the case of the EPA cell data, the first blot contained samples from time 0 to 60 minutes of treatment and the second from time 120 to 420 minutes.

**Table S1:**

| Reagent | Stock Concentration | Final Concentration | Solvent: |
|---|---|---|---|
| Diphenyleneiodonium (DPI) | Sigma Aldrich D2926 - 10mg | 1- 15 μM | DMSO |
| N-acetylcysteine (NAC) | Sigma Aldrich A9165-5 mg | 2 - 6 mM | $H_2O$ |
| VAS2870 | Enzo Life Sciences BML–EI395-0010 -10 mg | 5 - 20 μM | DMSO |
| U0126 | Sigma Aldrich | 1-40 μM | DMSO |

**Table S2: qRT-PCR zebrafish (*zf*) and *P.andruzzii* cavefish (*cf*) primers sequences**

| ***Gene:*** | **Forward primer** | **Reverse primer** |
|---|---|---|
| *zfβ-actin* | F: GCCTGACGGACAGGTCAT | R: ACCGCAAGATTCCATACCC |
| *zfper2* | F: CTTCACCACACCATACAGG | R: GTCTGACGGGGACGAGTCT |
| *zfcry1a* | F: TCCGCTGTGTGTACATCCTC | R: CAAACACTGCAGCAAAAACC |
| *cfcry1a* | F: GGCTCCACGACAATCCTTCA | R: TGGGGAAGACATCGGTAGGT |
| *cfper2* | F: CCGCAAAGTTTCCTTCGTCA | R: CATTACTGCCCAGACTCCCA |
| *cfβ-actin* | F: GATGAGGAAATCGCTGCCCT | F: GTCCTTCTGTCCCATGCCAA |

**Table S3: Antibodies**

| | |
|---|---|
| Sigma-Aldrich V9131 **α- vinculin** | α –vinculin monoclonal clone hVIN-1 |
| Cell Signaling 9211S **α- Phospho-p38 MAPK** | Thr180/Tyr182 |
| Cell Signaling 9101S **α- Phospho-p44/p42 MAPK** | Thr202/Tyr204 |
| Cell Signaling 9251S **α- Phospho SAPK/JNK** | T183/Y185 |

**Table S4: Luciferase reporters and expression constructs.**

| | |
|---|---|
| ***zf per1b-Luc*** | Vallone, Gondi et al. 2004 [25] |
| ***D-box$_{cry1a}$-Luc*** | Mracek et al. PLOS One, 2012 [26] |
| ***pcDNA3.1/myc-His/lacZ*** | Invitrogen |
| ***pGL3 Control*** | Promega |
| ***EGFP*** | *pCS2 EGFP* was a gift from Prof. Steffen Scholpp (University of Exeter, UK) |
| ***DN-p38*** | *pcDNA-3 Flag p38 alpha (agf)* a gift from Roger Davis (Addgene plasmid #20352) Enslen, Raingeaud et al. 1998 [66] |

| | |
|---|---|
| ***DN-JNK1*** | *pcDNA-3 Flag Jnk1a1(apf)* a gift from Roger Davis (Addgene plasmid # 13846) Derijard, Hibi et al. 1994 [67] |
| ***TEF1*** | *pcDNA3.1-TEF1* Vatine et al. PLOS Biology, 2009 [37] |
