# Supplementary material for: Evolution shapes the responsiveness of the D-box enhancer element to light and reactive oxygen species in vertebrates
Source: Sci Rep. 2018 Sep 4;8:13180. doi: 10.1038/s41598-018-31570-8 (PMC6123470; doi:10.1038/s41598-018-31570-8)
Supplement: Supplementary file 2 — Original western blot images [file 41598_2018_31570_MOESM2_ESM.pdf]

Evolution shapes the responsiveness of the D-box enhancer element to light and reactive oxygen species in vertebrates.

Cristina Pagano, Rima Siauciunaite, Maria L. Idda, Gennaro Ruggiero, Rosa M. Ceinos, Martina Pagano, Elena Frigato, Cristiano Bertolucci, Nicholas S. Foulkes and Daniela Vallone

Original western blotting data

Original western blotting data for Figure S3

Original western blotting data from **HeLa** cells treated in a time course of 420 minutes with **300 µM H_2O_2**. The antibody used and in which main figure the cropped images are included, are indicated in each panel.

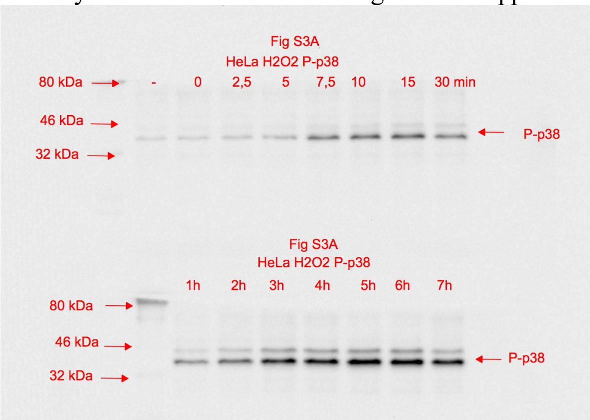

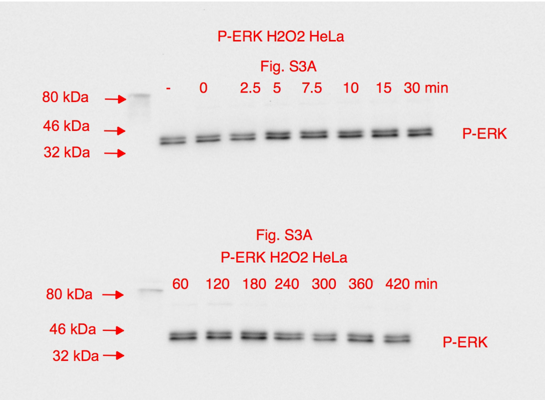

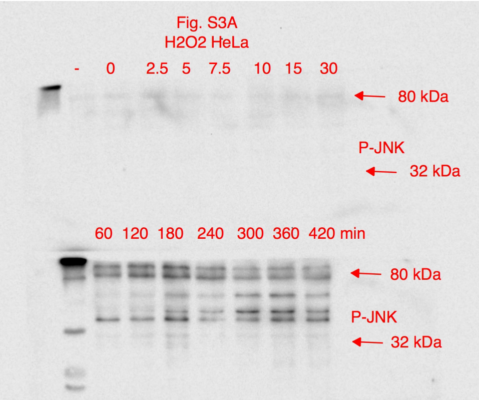

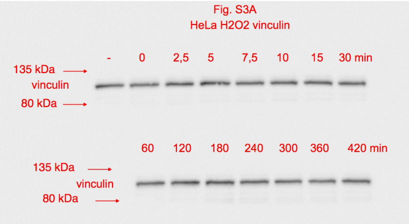

Original western blotting data from **PAC-2 cells** treated in a time course of 420 minutes with **300 µM H_2O_2**. The antibody used and in which main figure the cropped images are included, are indicated in each panel.

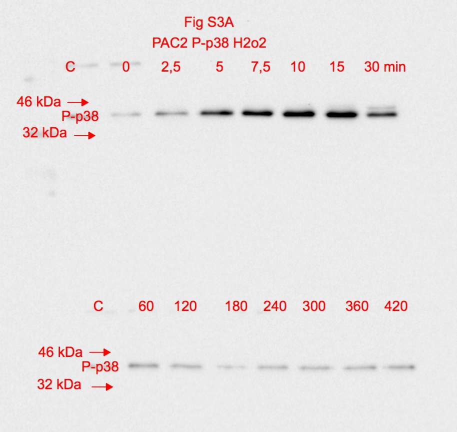

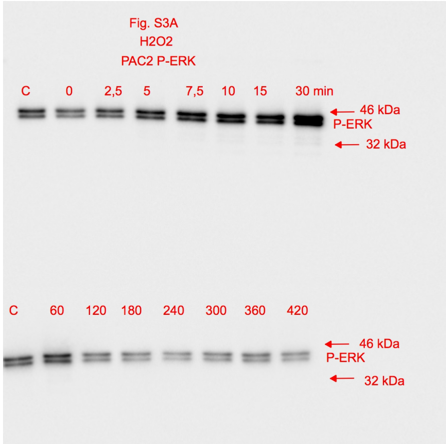

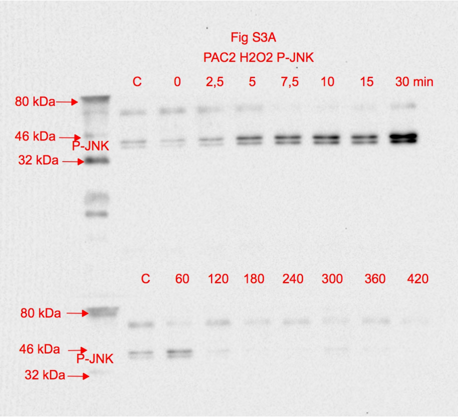

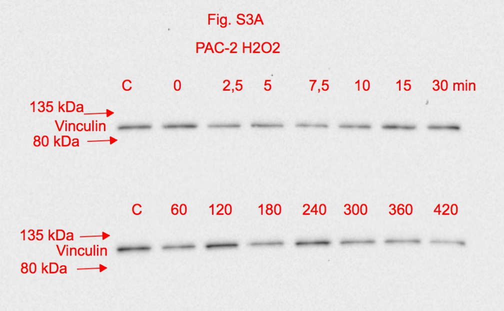

Original western blotting data from **EPA cells** treated in a time course of 420 minutes with **300 µM H_2O_2**. The antibody used and in which main figure the cropped images are included, are indicated in each panel.

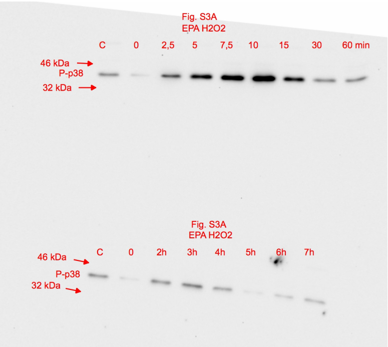

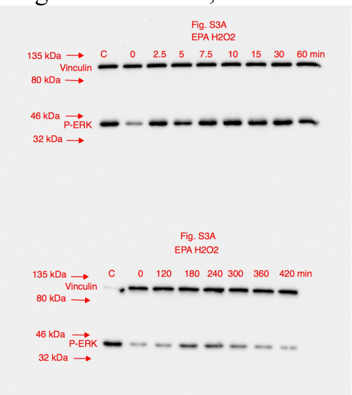

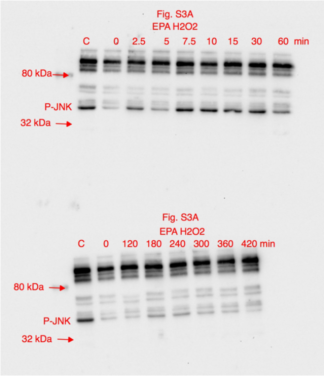

Original western blotting data from **HeLa cells** exposed to **blue light** in a time course of 420 minutes. The antibody used and in which main figure the cropped images are included, are indicated in each panel.

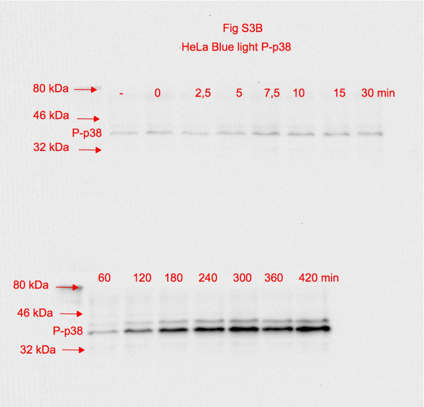

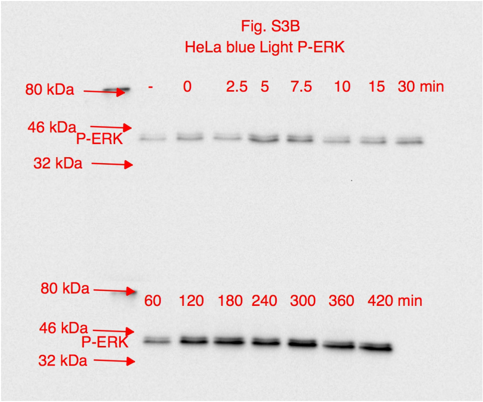

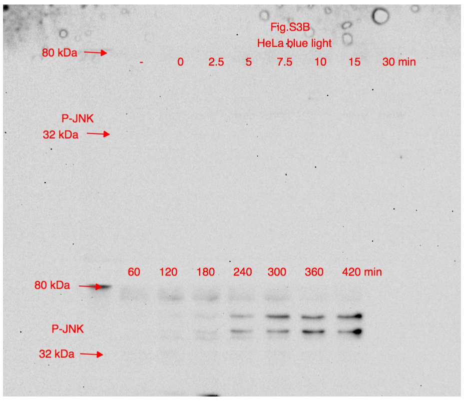

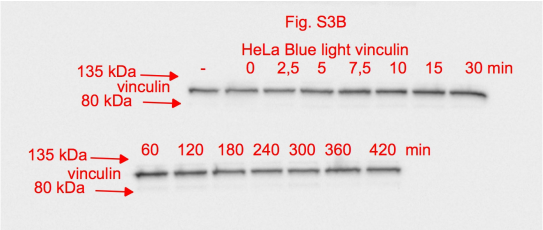

Original western blotting data from **PAC-2 cells** exposed to **blue light** in a time course of 420 minutes. The antibody used and in which main figure the cropped images are included, are indicated in each panel.

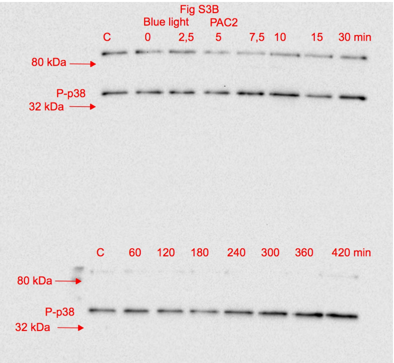

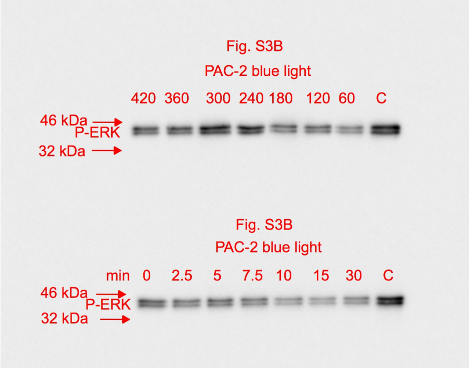

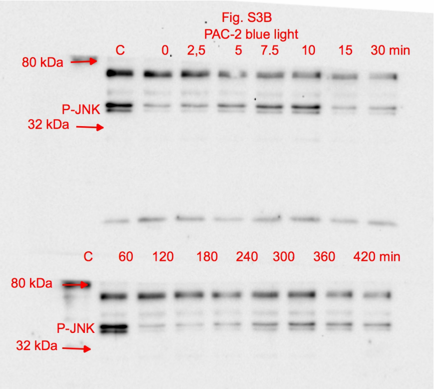

Original western blotting data from **EPA cells** exposed to **blue light** in a time course of 420 minutes. The antibody used and in which main figure the cropped images are included, are indicated in each panel.

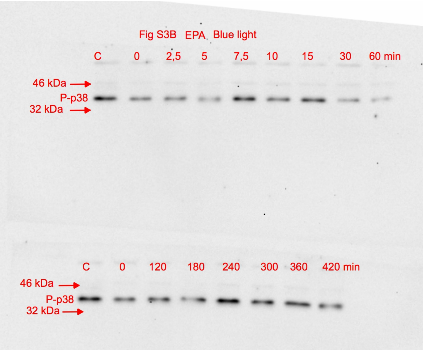

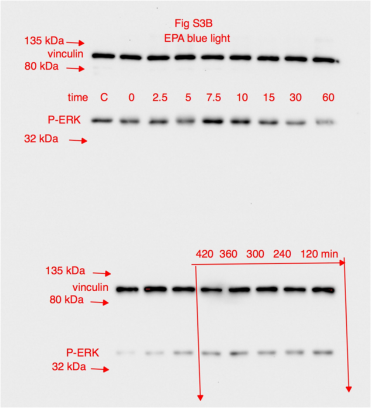

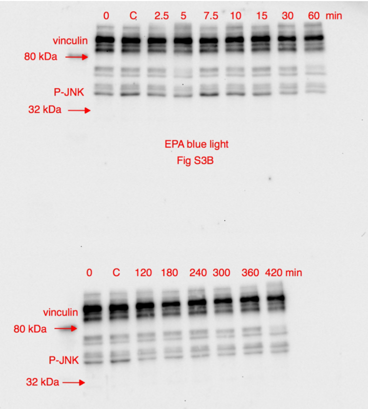

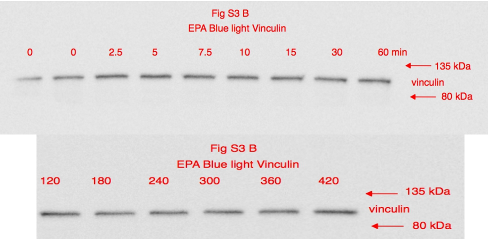

Original western blotting data for Figure 4

Original western blotting data from **PAC-2 cells** treated in a time course of 180 minutes with **300 µM H_2O_2**. The antibody used and in which main figure the cropped images are included, are indicated in each panel.

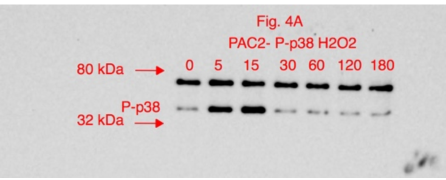

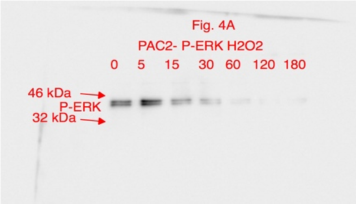

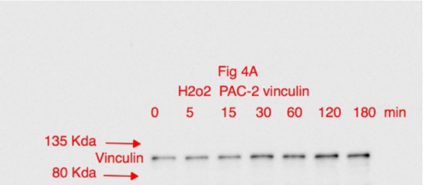

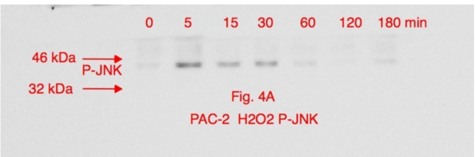

Original western blotting data from **PAC-2 cells** exposed to **blue light** in a time course of 180 minutes. The antibody used and in which main figure the cropped images are included, are indicated in each panel.

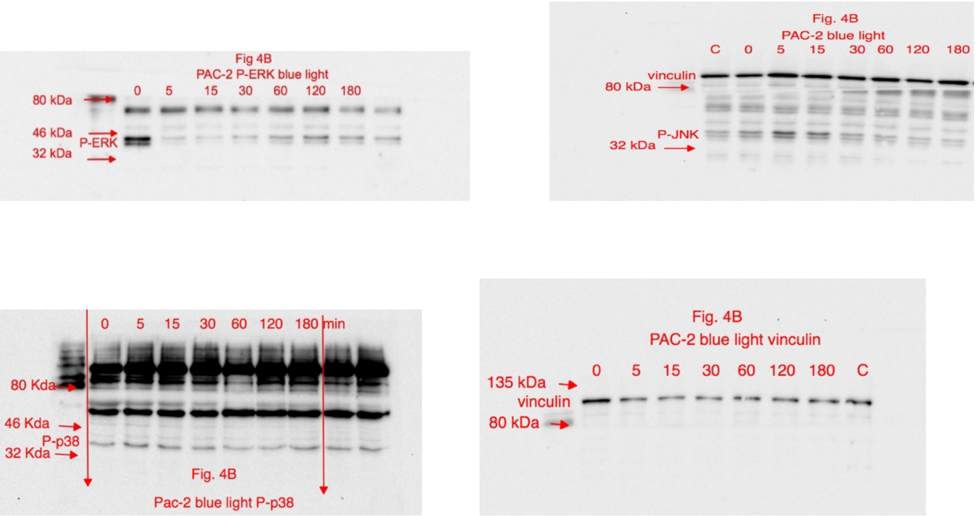

Original western blot of **PAC-2** cells exposed for 60 minutes to **blue light** and treated with the two ROS inhibitors VAS and NAC. The antibody used and in which main figure the cropped images are included, are indicated in each panel.

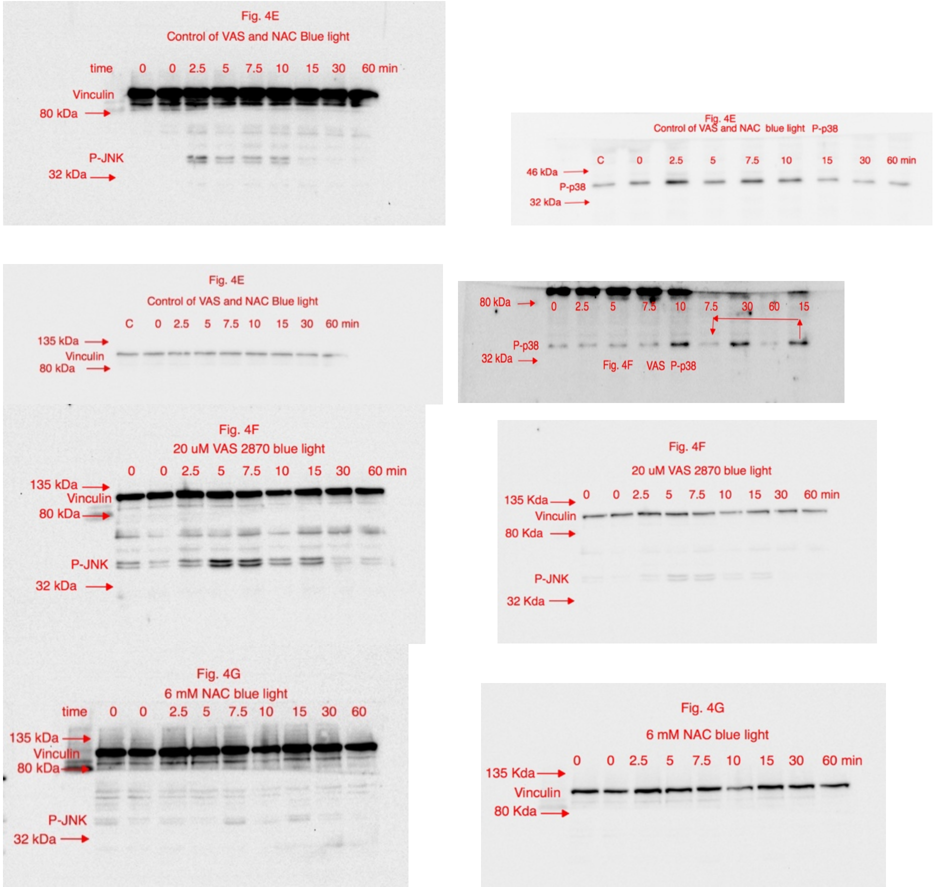

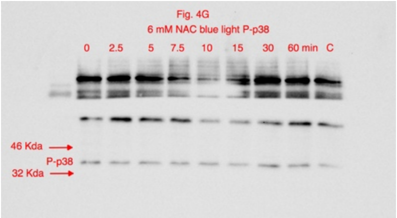
Fig. 4G
6 mM NAC blue light P-p38
0
2.5
5
7.5
10
15
30
60 min
C
46 Kda
P-p38
32 Kda
